# Carvedilol Exerts Neuroprotective Effect on Rat Model of Diabetic Neuropathy

**DOI:** 10.3389/fphar.2021.613634

**Published:** 2021-04-13

**Authors:** Rania M. Magadmi, Mujahid A. Alsulaimani, Aziza R. Al-Rafiah, Muhammad Saeed Ahmad, Ahmed Esmat

**Affiliations:** ^1^ Pharmacology Department, Faculty of Medicine, King Abdulaziz University, Jeddah, Saudi Arabia; ^2^ Neuroscience Unit, Faculty of Medicine, King Abdulaziz University, Jeddah, Saudi Arabia; ^3^ Department of Pharmacy, Ministry of Health, Taif, Saudi Arabia; ^4^ Medical Laboratory Technology Department, Faculty of Applied Medical Sciences, King Abdulaziz University, Jeddah, Saudi Arabia; ^5^ King Fahad Medical Research Center, King Abdulaziz University, Jeddah, Saudi Arabia; ^6^ Department of Pharmacology and Toxicology, Faculty of Pharmacy, Ain Shams University, Cairo, Egypt

**Keywords:** diabetes, carvedilol, diabetic neuropathy, antioxidants, in vivo

## Abstract

Diabetic neuropathy (DN) commonly occurs in diabetics, affecting approximately 50% of both type 1 and 2 diabetic patients. It is a leading cause of non-traumatic amputations. Oxidative stress could play a key role in the pathophysiology of DN. This study aimed to investigate the potential neuroprotective effect of carvedilol on STZ-induced DN in rats. Thirty male Sprague Dawley rats (weighing 200–250 g) were randomly divided into five groups (six/group), where group 1 (negative control) received only the vehicle (0.5% of carboxymethyl cellulose orally 1 ml/kg). DN was induced by a single injection of remaining rats with streptozotocin (STZ; 50 mg/kg, i.p.). After diabetes induction, group 2 served as the diabetic untreated animals; while groups 3 and 4 were treated with carvedilol (1 and 10 mg/kg/d, orally, respectively). Group 5 received *a*-lipoic acid as a reference neuroprotective (100 mg/kg/d, orally). All treatments were continued for 45 days after diabetes induction, followed by behavioural tests. After sacrificing the animals, dorsal root ganglia, and sciatic nerves were collected for histopathological examination and biochemical assessments. Briefly, STZ administration caused cold allodynia, induced oxidative stress, and increased nerve growth factor (NGF) concentration. Nevertheless, carvedilol improved the behavioural tests, ameliorated the oxidative imbalance as manifested by reducing malondialdehyde, restoring glutathione content, and superoxide dismutase activity. Carvedilol also decreased NGF concentration in DRG homogenate. In conclusion, this study demonstrates the neuroprotective effect of carvedilol in an experimentally induced DN rat model through–at least partly–its antioxidant effect and reduced NGF concentration in DRG.

## Introduction

Diabetes mellitus (DM) is an endocrine disorder that is characterized by hyperglycemia in response to absent or inadequate insulin secretion or impairment of insulin action (Katzung, 2014). It is a major health problem worldwide (Tabish, 2007), with a prevalence of 382 million people and nearly 5.1 million deaths in 2013 (International Diabetes Federation, 2013). Globally, the Middle East, especially the Gulf region, have the highest incidence rates of diabetes, where around 34 million (9.2%) people are currently living with DM; this number is predicted to reach around 67.9 million (11.6%) in 2035 (Majeed et al., 2014). In Saudi Arabia, diabetes is prevalent in 24% of its population (International Diabetes Federation, 2013), placing it among the top 10 countries with the highest prevalence rate worldwide (Alzahrani et al., 2015).

The impact of both hyperglycemia and vascular impairment results in neuronal dysfunction in the peripheral nervous system (Rahimi-Madiseh et al., 2016). Diabetic neuropathy (DN) is heterogeneous, affecting multiple parts of the nervous system with different symptoms (Boulton et al., 2005). Boulton *et al.* (Boulton et al., 1998) defined DN as ‘the presence of symptoms and/or signs of peripheral nerve dysfunction in people with diabetes after the exclusion of other causes’. DN is a common occurrence, which affects nearly 50% of patients with diabetes (Tesfaye, 2011).

Several mechanisms could elucidate the pathogenesis of hyperglycemia to induce microvascular complication in diabetic patients. Oxidative stress could play a major role in cellular injury (Fowler, 2008). A small variation in basal glucose levels could induce reactive oxygen species (ROS), resulting in neuronal injury (Russell et al., 2002). Some *in vitro* and *in vivo* studies demonstrated that an increase in glucose level leads to the overproduction of oxidative stress biomarkers, such as malondialdehyde (MDA) and advanced glycation end products, along with the inhibition of endogenous antioxidant synthesis (Cederberg et al., 2001).

Currently, the critical aspect of DN management is primarily attained by maintaining blood glucose within the normal range and adopting symptomatic therapy (Callaghan et al., 2012). Despite comprehensive research on managing DN-related chronic pain, limited success has been recorded, and the clinical needs remain mostly unmet (Kumar and Mittal, 2017). Hence, more research is warranted to discover novel and more effective medication, which can prevent the progression of DN and associated pain (Tesfaye, 2011).

Carvedilol is a non-selective *β*-adrenoreceptor blocker, which exerts a peripheral vasodilatory effect by blocking the *α*1-adrenoreceptor (Ruffolo et al., 1990). It is commonly used as an antihypertensive drug and in the treatment of chronic heart failure (Nichols et al., 1989). Recently, carvedilol has been shown to exhibit antioxidant activity (Pereira et al., 2011; Arab and El-Sawalhi, 2013; Yasar et al., 2013; Diogo et al., 2017) and counteract the oxaliplatin-induced oxidative stress in neuronal cells (Areti et al., 2017). Besides, carvedilol prevents reduction in the nerve conduction velocity, secondary to a reduction in nerve perfusion in streptozotocin (STZ)-induced diabetic rats (Cotter and Cameron, 1995). Furthermore, the antioxidant activity of carvedilol blocks the ability of oxygen radicals from damaging coronary arteries dilated through the action of nitric oxide (Ruffolo and Feuerstein, 1997).

Diabetes and hypertension are common comorbidities in the elderly (Aronow, 2008); the incidence of hypertension increases among diabetic patients (Sowers et al., 2001) and vice versa (Gress et al., 2000). Over, the attractive properties of carvedilol make it a suitable therapeutic candidate for managing DN. Hence, this study aimed to investigate the potential neuroprotective effect of carvedilol on STZ-induced DN in rats.

## Materials and Methods

### Ethics Statement

Animal handling and all *in vivo* procedures adhered to the institutional ethical guidelines. This study protocol was approved in advance by the Bioethical Committee of our institute (Reference No. 237-18). Of note, all efforts were made to reduce the suffering of experimental animals. Study carried out in accordance with National Institutes of Health guide for the care and use of Laboratory animals (NIH Publications No. 8023, revised 1978).

### Study Population

Thirty male Sprague Dawley adult rats (weight, 200–250 g) were purchased, and housed them at the animal house of King Fahd Medical Research Center, King Abdulaziz University, Jeddah, Saudi Arabia. All animals were housed at three per cage, with free access to food and water. During the experimentation, a constant temperature of 23 ± 1°C, relative humidity 55 ± 10% and 12:12 h light–dark cycle were maintained.

### Experimental Study Design, Diabetes Induction and Treatment Protocols

Thirty rats were randomly divided into five groups (6 rats/group), where group 1 (negative control) received only the vehicle (0.5% of carboxymethyl cellulose CMC, orally). Diabetes was induced in groups 2–5 using a single intraperitoneal (i.p.) injection of STZ [Sigma Aldrich (MERCK), United States] at 50 mg/kg (Srinivasan and Ramarao, 2007). Only animals with fasting blood glucose (FBG) more than 250 mg/dl have been included. Group 2 served as diabetic untreated animals; whereas groups 3 and 4 were treated with carvedilol [1 and 10 mg/kg/d, orally), respectively], (Ruffolo and Feuerstein, 1997). Group 5 received α-lipoic acid as a reference neuroprotective (100 mg/kg/d, orally). Selection of α-lipoic acid as a reference was based on its potent antioxidant activity in ROS scavenging and regeneration of endogenous antioxidants compounds (Oyenihi et al., 2015). Furthermore, it has been reported to improve the nerve blood flow and nerve conduction velocity (Papanas and Ziegler, 2014). All treatments were performed for 45 days after diabetes induction.

**FIGURE 1:**
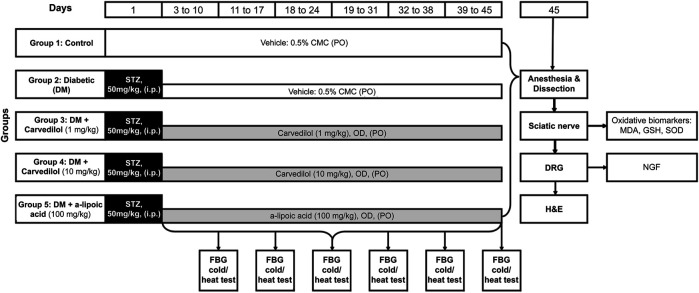
Structural summary of the study design.

Fasting blood glucose, animals’ body weights (BW) and the time latency for heat and cold stimuli were all assessed weekly after STZ administration as indicators for DN. Thereafter, rats were euthanized using 1.2 g/kg of urethan single i.p. injection. Urethan was used because it is recommended when the preservation of neuronal transmission is required (Field et al., 1993). Then, the sciatic nerves were isolated from the two hind limbs and weighed for each rat separately. The spinal cords of all rats were also dissected and–under the microscope (Optika, T1A250V, Italy)–each spinal cord was dissected longitudinally into two halves, following which the dorsal root ganglion (DRG) was collected and weighed. The sciatic nerve and DRG tissues were frozen at −80°C until homogenization and biochemical analyses. Representative DRG were fixed in 10% formalin/saline for histopathological examination. Figure 1 summarizes the study design of current study.

### Fasting Blood Glucose

Fasting blood glucose was measured using a glucometer (Accu Chek performa, Roach, Switzerland). Blood samples were collected from the tail vein of the fasting animals. The rats were considered a diabetic when blood glucose levels were above than 250 mg/dl.

### Behavioral Assessment

DN pain was assessed by measuring cold allodynia and hot hyperalgesia using analgesiometer (model 35100; Ugo Basile, Italy) every week. Of note, all the studies were analyzed by a single-blinded observer. The temperature of cold allodynia experiment was set at 21°C. The hot hyperalgesia response was measured using a hot plate analgesia meter (Ugo Basile, Italy); the plate temperature was set at 48°C. In this study, the latency period was defined as the time from when an animal was placed on the plate until it licked its paw or tried to jump or any abnormal response to avoid this exposure (Di Cesare Mannelli et al., 2014). The maximum exposure time was 40°s to protect the animal from tissue injury. All rats were assessed two times, with 5°min intervals.

### Histopathological Examination

After fixation in 10% formalin/saline for 24 h, representative DRGs were transferred to suitable cassettes. Then, the cassettes were processed over-night to prepare the tissue for paraffin embedding using specialized processing machine (TP1020, Leica Semi-enclosed Benchtop Tissue processor, Germany). On the next day, the cassettes were transferred to paraffin embedding machine (EG1160, Feica, Germany) to prepare paraffin blocks. After 24 h, tissue slides (5 µm thickness) were prepared from the solidified blocks using Accu-Cut^®^ microtome SRM 200 (SAKURA, Netherland). Subsequently, all samples were put in the automated machine YABO-700 (China) for hematoxylin and eosin staining. After that, slides were mounted and covered with coverslips, then left until drying.

### Biochemical Analyses

The sciatic nerve was homogenized in cold phosphate-buffered saline (PBS) in 1:10 ratio to determine the oxidative stress biomarkers. Typically, the tissue was diluted by mixing 1 mg of the sciatic nerve and 10 μL of PBS. Next, the tissue was stored at −40°C for a freeze–thaw cycle to break the cell membranes further. Then, the TissueLyser apparatus (TissueLyser II; QIAGEN, Hilden, Germany) was used at the frequency of 25/s for 90 s. The supernatant was collected after centrifuging the sample at 5000 rpm for 15 min, which was used for the analyses. In addition, commercial enzyme-linked immunosorbent assay (ELISA) kits were utilized to assess MDA concentraion, GSH content and SOD activity (MyBioSource, San Diego, California, United States). Furthermore, the nerve growth factor (NGF) was measured using the Rat NGF ELISA kit (MyBioSource). All procedures were performed per manufacturers’ protocols.

### Statistical Analysis

The results were presented as mean ± standard deviation (SD). FBG data and biochemical data were analyzed using one-way analysis of variance (ANOVA), followed by Tukey’s post-hoc test. In addition, the data obtained from behavioral tests were analyzed using two-way ANOVA. *p* value of <0.05 was considered as statistically significant. In this study, all statistical analyses were performed using GraphPad InStat version 3; the graphs were obtained using GraphPad Prism version 8 (GraphPad Software, La Jolla, CA).

## Results

### Fasting Blood Glucose and Body Weight

Table 1 shows the FBG levels in different treatment groups at weeks 1–4 after diabetes induction. The FBG levels of all treatment groups were significantly elevated compared with the corresponding control at each week. However, treatment with carvedilol (1 and 10 mg/kg/d) failed to cause any significant decrease of FBG levels, compared to the corresponding diabetic group. One the other hand, animals treated with α-lipoic acid had significantly reduced FBG by approximately 28 and 35% at the third and fourth weeks, respectively, compared with the corresponding diabetic group. A similar pattern was observed with animals’ body weights, as shown in Table 2. At week 1, minor nonsignificant changes were detected in all groups compared to the corresponding control. Starting from week 2, BW of all groups started to decrease significantly from the corresponding control group. In comparison to diabetic group, treatment with carvedilol (1 and 10 mg/kg/d) was unable to significantly prevent BW loss, however, lipoic acid treatment was more effective and caused a significant increase in body weight. These effects were deepened at the last week, where STZ significantly reduced BW by about 35% compared to the corresponding control, and treatment with carvedilol (1 and 10 mg/kg/d) did not significantly improve BW from the diabetic group. Yet, treatment with lipoic acid significantly increased BW by about 33%, compared to STZ-induces diabetes group.

**TABLE 1:** Effect of carvedilol on FBG levels in STZ-induced diabetes in rats.

*FBG Group*	Control	DM	DM + carvedilol (1 mg/kg/d)	DM + carvedilol (10 mg/kg/d)	DM + lipoic acid (100 mg/kg/d)
Week 1 FBG (mg/dl)	84.16 ± 7.03	465.3^a^ ± 71.87	395.2^a^ ± 37.81	380.6^a^ ± 56.31	433^a^ ± 84.94
Week 2 FBG (mg/dl)	82.7 ± 5.15	411^a^ ± 12.73	358.5^a^ ± 18.77	443.25^a^ ± 86.47	398.25^a^ ± 64.57
Week 3 FBG (mg/dl)	86.7 ± 5.13	480.6^a^ ± 55.23	386.7^a^ ^,^ ^b^ ± 36.89	441.3^a^ ± 54.02	345.8^a^ ^,^ ^b^ ± 30.43
Week 4 FBG (mg/dl)	84.22 ± 6.89	499.6^a^ ± 53.63	437.3^a^ ± 25.63	443.2^a^ ± 45.17	322.8^a^ ^,^ ^b^ ± 35.11

Data presented as mean ± SD (*n* = 6).

DM, diabetes mellitus; FBG, fasting blood glucose; SD, standard deviation; STZ, streptozotocin.

Statistical analysis was accomplished by one way ANOVA, followed by Tukey’s post-hoc test.

^a^ Statistically significant from the corresponding control group at *p* < 0.05.

^b^ Statistically significant from the corresponding diabetes group at *p* < 0.05.

**TABLE 2:** Effect of carvedilol on body weight in STZ-induced diabetes in rats.

*BW Group*	Control	DM	DM + carvedilol (1 mg/kg/d)	DM + carvedilol (10 mg/kg/d)	DM + lipoic acid (100 mg/kg/d)
Week 1 BW (g)	227 ± 12.2	205.5 ± 10.7	206.2 ± 11.7	215.3 ± 13.5	213.5 ± 15.5
Week 2 BW (g)	246.8 ± 13.3	192.2^a^ ± 9.5	198.2^a^ ± 12.2	204.7^a^ ± 13.2	217.3^a^ ^,^ ^b^ ± 15.8
Week 3 BW (g)	255.7 ± 14.5	180.7^a^ ± 8.4	189.2^a^ ± 13.9	199.3^a^ ± 13.7	223.5^a^ ^,^ ^b^ ± 16.6
Week 4 BW (g)	263.2 ± 15.1	171.7^a^ ± 7.3	184.5^a^ ± 12.5	186.2^a^ ± 14.2	228.8^a^ ^,^ ^b^ ± 17.4

Data presented as mean ± SD (*n* = 6).

DM, diabetes mellitus; BW, Body weight; SD, standard deviation; STZ, Streptozotocin.

Statistical analysis was accomplished by one-way ANOVA, followed by Tukey’s post-hoc test.

^a^ Statistically significant from the corresponding control group at *p* < 0.05.

^b^ Statistically significant from the corresponding diabetes group at *p* < 0.05.

### Effect of Carvedilol on Behavioral Tests

**FIGURE 2:**
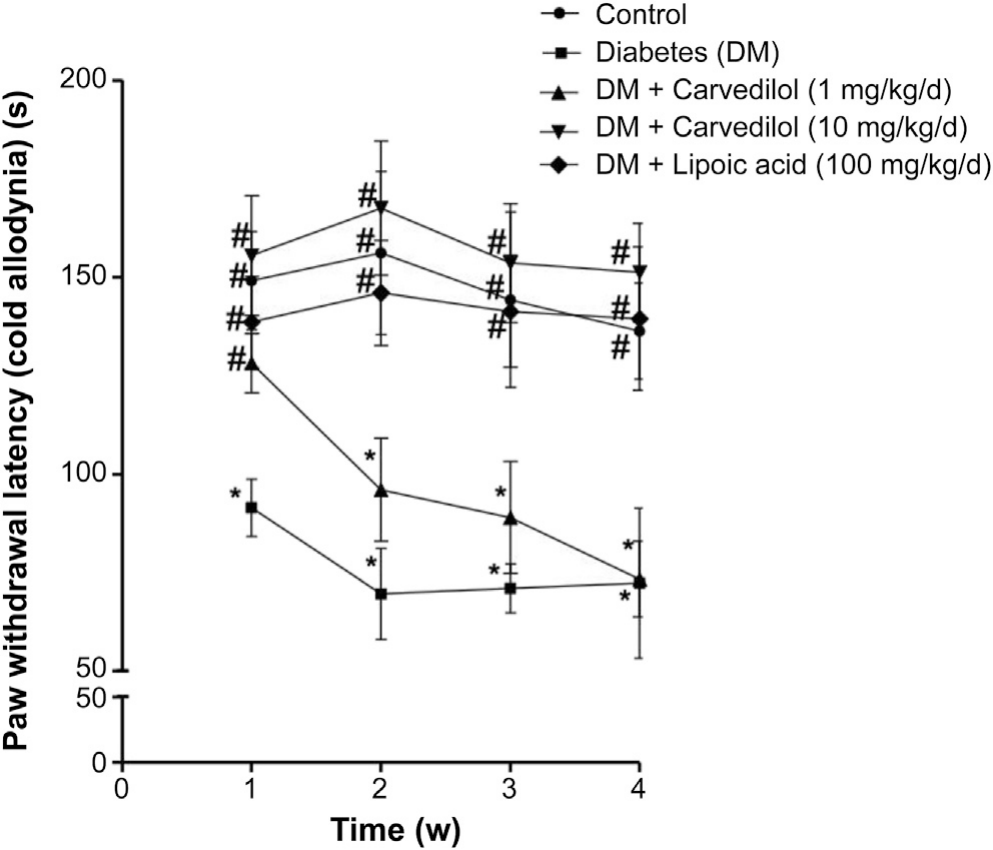
The paw withdrawal latency to cold stimuli in STZ-induced diabetes in rats. Data are presented as mean ± SD for each group (*n* = 6). Statistical analysis was done by two-way ANOVA, followed by Tukey’s post-hoc test. *Statistically significant from the corresponding control group (group 1) at *p* < 0.05. #Statistically significant from the corresponding diabetes group at *p* < 0.05. DM, diabetes mellitus; SD, standard deviation; STZ, streptozotocin; Time (w), time in weeks.

**FIGURE 3:**
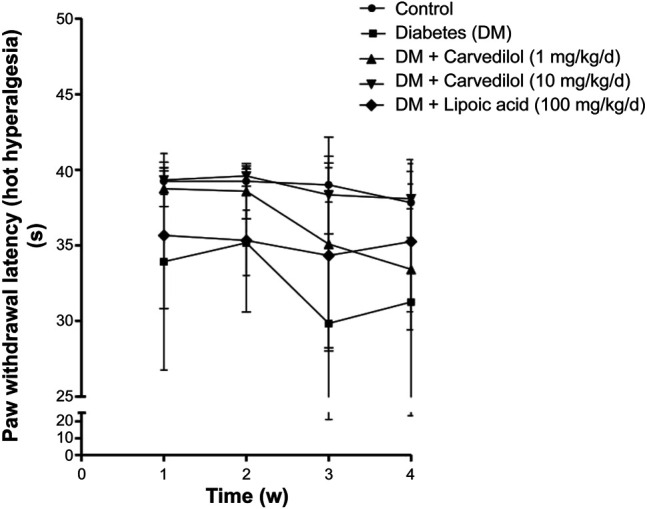
The paw withdrawal latency to hot stimuli in STZ-induced diabetes in rats. Data are presented as mean ± SD for each group (*n* = 6). DM, diabetes mellitus; SD, standard deviation; STZ, streptozotocin; Time (w), time in weeks. Statistical analysis was done by two-way ANOVA.

Figure 2 shows a significant decline (*p* < 0.001) in the paw withdrawal latency to cold stimuli (allodynia) in the diabetic group (group 2), compared with the corresponding control, starting from week 1 of the experiment. Carvedilol at high dose (10 mg/kg/d) was neuroprotective, manifested by a significant increase in the withdrawal latency to cold stimuli, compared with the diabetic group (group 2) throughout the study. Remarkably, the neuroprotective effect of high-dose carvedilol was comparable to that manifested by α-lipoic acid. Conversely, low-dose carvedilol (1 mg/kg/d) was neuroprotective only during week 1 and failed to exhibit any significant elevation of the withdrawal latency compared with the diabetic group (group 2) during the remaining experimental period. Compared with the control group (group 1), the latencies of the animals’ responses to hot stimuli were not significantly decreased in the diabetic group (group 2). Carvedilol caused a dose-related enhancement in latency time to hot stimuli from the diabetic group (group 2); however, these changes were statistically non-significant (*p* > 0.05). Furthermore, the effect of carvedilol at both doses was non-significant from that of *α*-lipoic acid (Figure 3).

### Effect of Carvedilol on STZ-Induced Oxidative Stress Biomarkers

#### Effect of Carvedilol on the MDA Concentration

**FIGURE 4:**
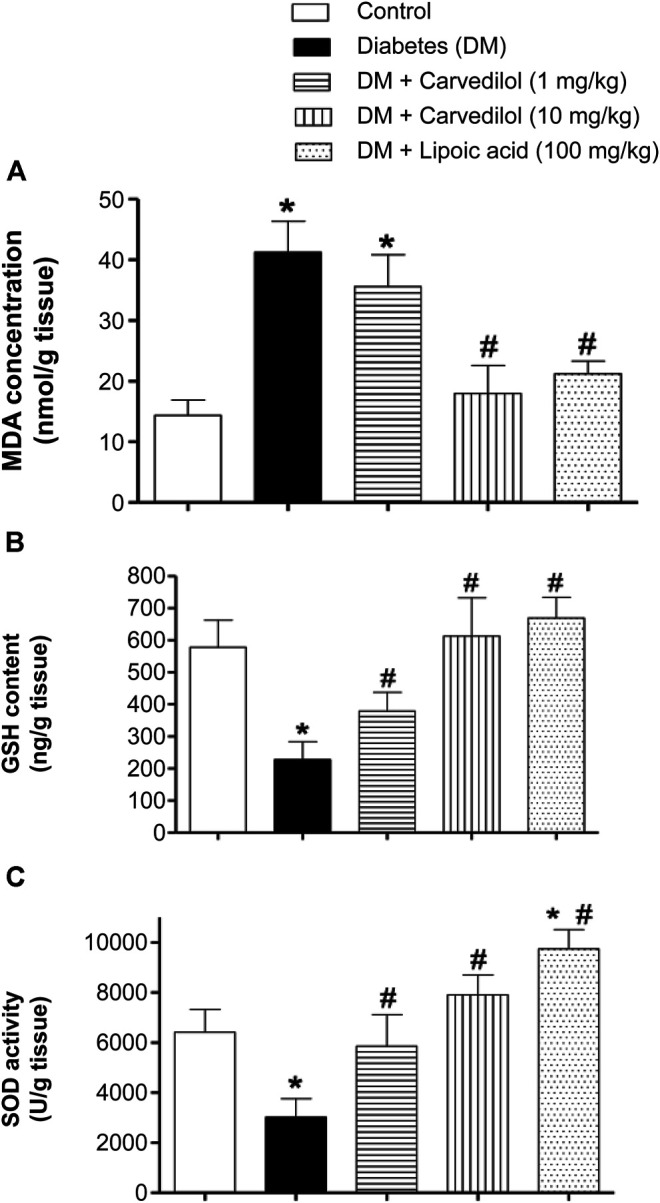
Effect of carvedilol on STZ-induced oxidative stress in the sciatic nerve of diabetic rats. **(A)** The MDA concentration in the sciatic nerve. **(B)** The GSH content in the sciatic nerve. **(C)** The SOD activity in the sciatic nerve. Data are presented as mean ± SD for each group (*n* = 6). *Statistically significant from the corresponding control group at *p* < 0.05. #Statistically significant from the corresponding diabetes group at *p* < 0.05. DM, diabetes mellitus; GSH, glutathione; MDA, malondialdehyde; SD, standard deviation; SOD, superoxide dismutase; STZ, streptozotocin.

Figure 4A shows the MDA concentration as an indicator for lipid peroxidation in STZ-induced DM in rats. DM induction in animals (group 2) caused more than twofold increase in the MDA concentration, which was statistically significant (*p* < 0.001), compared with the control group (group 1). Regrettably, low-dose carvedilol (1 mg/kg/d) (group 3) failed to significantly reduce the MDA concentration, compared with the diabetic group (group 2; *p* = 0.11). However, high-dose carvedilol (10 mg/kg/d) (group 4) ameliorated the MDA concentration elevation, causing a significant reduction (*p* < 0.001) by approximately 56% from the diabetic group (group 2). Likewise, treatment with *α*-lipoic acid (group 5) significantly (*p* < 0.001) decreased the MDA concentration by nearly 48%, compared with the diabetic group (group 2) without any significant change from the control group (group 1).

#### Effect of Carvedilol on the GSH Content

In this study, STZ-induced diabetes (group 2) significantly depleted the sciatic nerve GSH content, decreasing it to about 40% of the control value (group 1) (*p* < 0.001) (Figure 4B). Besides, treating the animals with carvedilol (group 3 and 4) significantly ameliorated GSH depletion in a dose-related manner (*p* < 0.001). Notably, both doses of carvedilol restored the GSH content to the standard value of the control group (group 1). Similarly, α-lipoic acid (group 5) tremendously compensated the depleted GSH content, reaching even more than the control value (group 1).

#### Effect of Carvedilol on the SOD Activity

Figure 4C shows changes in the enzymatic activity of SOD. In the diabetic group (group 2), the SOD activity was markedly decreased by >50%, compared with the control group (group 1). Conversely, carvedilol distinctly enhanced the SOD activity at both doses (1 and 10 mg/kg/d), compared with the diabetic group (group 2); this effect exceeded the control value with carvedilol (10 mg/kg/d) (group 4) by approximately 23%. Interestingly, *α*-lipoic acid (100 mg/kg/d) (group 5) not only prevented any inhibition of the SOD activity resulting from diabetes but also significantly boosted the SOD activity by nearly 50%, above the control group (group 1).

### Histological Examination

**FIGURE 5:**
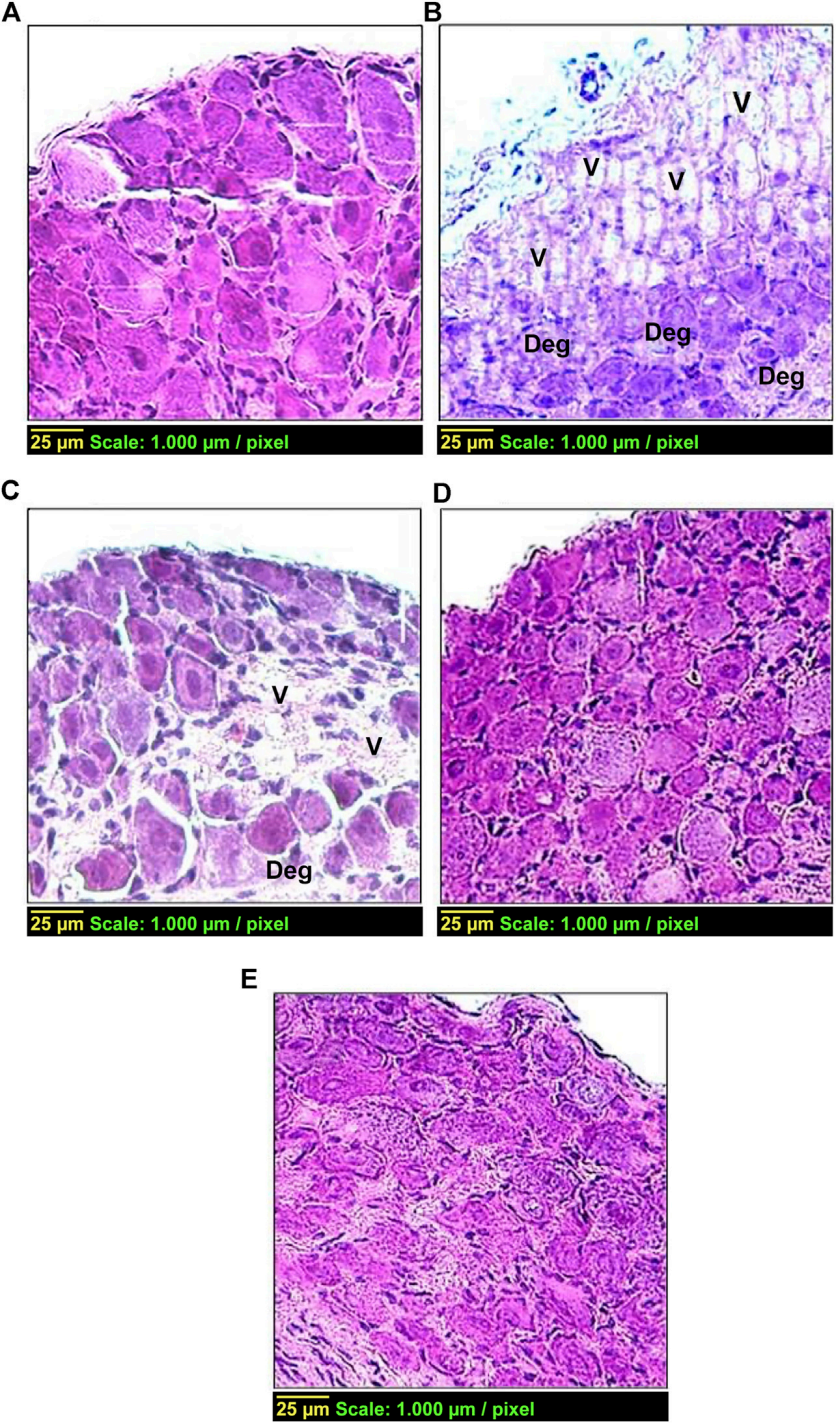
Effect of carvedilol on histopathological changes in the DRG tissue in STZ-induced diabetes in rats. **(A)** Control group with normal architecture. **(B)** Diabetic group with vacuolization ‘V’ (++++) and degeneration ‘Deg’ (++++) of the DRG tissue. **(C)** Diabetic rats treated with carvedilol (1 mg/kg/d) showing a lesser degree of pathological changes in the DRG tissue; ‘V’ +++ and ‘Deg’ +++. **(D)** Diabetic rats treated with carvedilol (10 mg/kg/d). **(E)** Diabetic rats treated with *a*-lipoic acid (100 mg/kg/d). Of note, both D and E had minimal pathological changes in the DRG tissue compared with the diabetic group.

Figure 5 shows histological changes in the DRG tissue from different treatment groups. The control group (group 1) (Figure 5A) displayed the standard histological architecture of the DRG tissue, while the diabetic group (group 2) exhibited a vacuolated, disorganized and degenerated tissue compared with the control group (group 1) (Figure 5B). These pathological changes were detected in group 3 (Figure 5C) but to a lesser extent than that in the diabetic group (group 2). Conversely, the histopathological changes were with minimal vacuolization and degeneration in groups 4 and 5, compared with the diabetic group (group 2) (Figures 5D,E, respectively).

### Effect of Carvedilol on the STZ-Increased NGF Content in the DRG Tissue.

**FIGURE 6:**
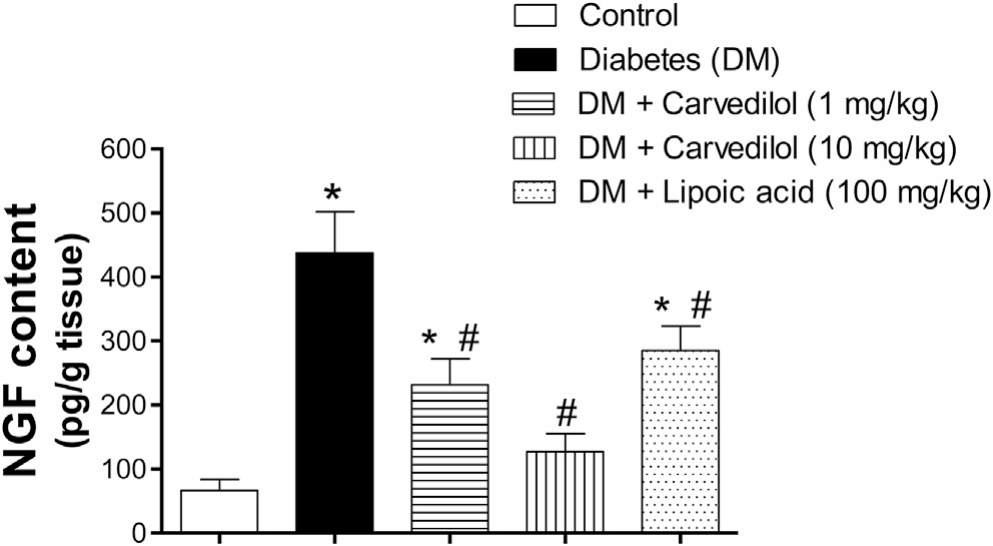
Effect of carvedilol on the NGF content in the DRG tissue of STZ-induced diabetes in rats. Data are presented as mean ± SD for each group (*n* = 6). *Statistically significant from the corresponding control group at *p* < 0.05. #Statistically significant from the corresponding diabetes group at *p* < 0.05. DM, diabetes mellitus; DRG, dorsal root ganglion; NGF, nerve growth factor; SD, standard deviation; STZ, streptozotocin.

Figure 6 shows the NGF content in the DRG. The NGF content in the diabetic group (group 2) was significantly elevated by more than sevenfold, compared with the control group (group 1). Compared with the diabetic group (group 2), carvedilol significantly reduced the abnormally elevated NGF content in the test groups (groups 3 and 4) in a dose-related manner. In addition, low-dose carvedilol exerted an effect similar to that of *α*-lipoic acid-treated group (group 5). Remarkably, high-dose carvedilol markedly reduced the NGF content to the extent that no significant difference was observed from the control group (group 1). Thus, high-dose carvedilol was superior to *α*-lipoic acid in reducing the effect of the NGF content in DRG.

## Discussion

Diabetic neuropathy (DN) is a common occurrence in patients with diabetes, affecting approximately 50% of both type 1 and 2 diabetic patients (Tabish, 2007). Saudi Arabia is ranked among the top 10 countries with the highest prevalence rate of diabetes worldwide (Alzahrani et al., 2015). Oxidative stress could play a key role in the pathophysiology of DN. The antihypertensive drug “carvedilol” is non-selective *β*-blocker with additional *a*-blocking activity. It has been shown to exhibit antioxidant activity (Pereira et al., 2011; Arab and El-Sawalhi, 2013; Yasar et al., 2013; Diogo et al., 2017) and prevent reduction of nerve conduction velocity (Cotter and Cameron, 1995). Most recently, carvedilol has been revealed by Magadmi et al. (Magadmi et al., 2021) to display a neuroprotective effect *in vitro* in high glucose-induced neuronal damage on cultured dorsal root ganglia. Hence, this work has been extended to investigate *in vivo* the potential neuroprotective effect of carvedilol on STZ-induced DN in rat model.

Basically, STZ is transported into pancreatic *β* cells via glucose transporter 2 (GLUT-2). Then, it causes destruction to *β* cells mainly through DNA alkylation, in addition to other factors like release of nitric oxide and generation of ROS (Radenkovic et al., 2016). According to various studies, STZ produces an early cold-allodynia and hot-hyperalgesia (Sharma et al., 2006a; Sharma et al., 2006b; Kuhad and Chopra, 2008; Ma et al., 2015; Areti et al., 2017) as well as a decrease in sensory nerve conduction velocities (Cotter and Cameron, 1995). These effects could be explained by the toxic effect of STZ and the prolonged exposure to hyperglycemia that results in excessive production of ROS causing destruction to the cell membrane, cell protein, and nucleic acid leading to cell death (Rahimi-Madiseh et al., 2016).

Concerning the effect of carvedilol, it was not able to exert any significant reduction of FGB levels, compared to the diabetic group at the corresponding week. This outcome is in parallel with Zheng et al. that investigated the effect of carvedilol on cardiac function in diabetic cardiomyopathy rats, where carvedilol treatment prevented all abnormal cardiac changes but without decreasing hyperglycemia (Liu et al., 2017). Thus, the protective effect of carvedilol against diabetic complications could be independent of any antihyperglycemic effect. This finding was also reflected on BW changes in the current study, where treatment with carvedilol did not prevent weight loss in diabetic animals. On the other hand, *α*-lipoic acid ameliorated the STZ-induced hyperglycemia and weight loss in the present study. These findings are in parallel with previous studies that explained the ability of *α*-lipoic acid to increase insulin sensitivity and blood glucose utilization in type 2 diabetic patients (Borcea et al., 1999; Evans and Goldfine, 2000; Evans et al., 2002) and prevent the glycation of some protein (Suzuki et al., 1992). Also, it could protect against the destruction of *β* cells in pancreas (Packer, 1998).

In the current study, there was a significant decrease in paw withdrawal latency to cold stimuli (allodynia) in the STZ-induced diabetic group, compared to the control animals, during the whole experimental period. In contrast, carvedilol at dose (10 mg/kg/d) was capable of attenuating this STZ painful effect from first to the fourth week of experiment. Concurrently, low-dose carvedilol (1 mg/kg/d) exhibited significant elevation of the withdrawal latency compared with the diabetic group (group 2) only at the first week of study. However, with the progress of the disease, low-dose carvedilol failed to improve the withdrawal latency.

Interestingly, it has been previously reported that carvedilol treatment significantly increased the paw withdrawal latencies to cold stimuli in oxaliplatin-induced neuropathy (Areti et al., 2017). In contrast, carvedilol caused a nonsignificant improvement in latency time to hot stimuli. This could be explicated by the complex challenges of studying pain in rodents and the associated variability of their responses to pain. Some of these variables include circadian rhythmicity, ambient temperature, bedding texture, cage density, and humidity in the laboratory (Mogil, 2009). More recently, it has been reported that exposure of mice and rats to male, but not female, experimenters produced pain inhibition, with stimuli emanating from males producing a robust physiological stress response resulting in stress-induced analgesia (Sorge et al., 2014).

The effect of carvedilol on the behavior experiments could be elucidated by its potent antioxidant activity. Thus, the effect of carvedilol on STZ-induced oxidative stress biomarkers was assessed. Currently, carvedilol has shown antioxidant properties, manifested by its ability to ameliorate the elevation of MDA concentration in sciatic nerve homogenate. Moreover, it restored the depletion of GSH content of sciatic nerve, compared with a diabetic group in a dose related manner. Likewise, carvedilol in both doses reversed the STZ effect on SOD activity, and was able to activate SOD to approach normal values. Indeed, sciatic nerve is the region of peripheral nervous system that is most affected by diabetic neuropathy. It has been indicated that hyperglycemia in STZ-induced DN leads to severe molecular defects, characterized by an outstanding upregulation of oxidative phosphorylation pathway in mitochondria as well as disturbances of lipid metabolism particularly in the sciatic nerve (Freeman et al., 2016).

In accordance with current findings, previous studies have demonstrated that carvedilol prevents depletion in the endogenous antioxidant (vitamin E and glutathione) in tissues subjected to oxidative stress *in vitro* and *in vivo* (Dandona et al., 2007)*.* Furthermore, another *in-vitro* study found that carvedilol protected PC12 cells from oxidative stress damage at concentrations 2.5–10 µM (Wang et al., 2014). Moreover, carvedilol caused significant reduction in the ROS in Neuro-2a (N2a) cells treated by oxaliplatin. and significantly ameliorated the elevation of MDA, nitrate, and restored the GSH content and ATP mitochondrial function in oxaliplatin treated rats (Areti et al., 2017). Addtionally, carvedilol (10 µM) was found to significantly attenuate glutamate and H_2_O_2_-induced cytotoxicity by decreasing the production of ROS in HT22 cells. However, it failed to reverse glutamate induced depletion of intracellular GSH level in HT22 cells (Ouyang et al., 2013). Another study showed that chronic administration of carvedilol significantly ameliorated the increase in MDA and ROS concentrations, while significantly prevented the depletion of GSH content in colchicine-treated rats (Kumar and Dogra, 2009). Earlier, in a clinical randomized double blind study on carvedilol and atenolol, MDA level was measured spectrophotometrically as the key marker of lipid peroxidation. The study showed a significant MDA reduction by 30% in patients received carvedilol while a non-significant change in patients treated with atenolol (Giugliano et al., 1997). Another clinical study has showed that carvedilol significantly reduced plasma 8-hydroxy-2-deoxyguanosine (hs8-OHdG) biomarker level, compared with both hydrochlorothiazide and control (Lee et al., 2000). Taking all together, these studies confirm the antioxidant activity of carvedilol on different tissues on cellular, animal, and human levels.

Mechanistically, carvedilol exerts its antioxidant activity through chemical and biological interactions. Carvedilol could bind chemically and scavenge free radicals. Biologically, carvedilol could inhibit the enzymes that generate the ROS, leading to marked decreased in the ROS production (Dandona et al., 2004). Moreover, carvedilol at (10 mg/kg, p.o.) has been shown to exert maximal antioxidant properties without any toxic manifestations (Sahu et al., 2014). Compared to vitamin E, carvedilol represents 10 times more potent than vitamin E activity. As well, the metabolite of carvedilol has 50–100 fold more potent than the parent compound and 1000-fold more potent than vitamin E (Dandona et al., 2007)*.* However, it is only beta blocker containing carbazole moiety which is responsible for the antioxidant activity of carvedilol (Yue et al., 1992; Yue et al., 1994; Ruffolo and Feuerstein, 1997). In this regard, *α*1 blocking activity of carvedilol cannot be excluded as a neuroprotective mechanism in DN. It had been previously suggested that increased *α*1-adrenoceptors in DRG–but not in the spinal cord–might participate in the pathogenesis of painful DN (Lee et al., 2000). In addition, *α*1‐blockers like prazocin could ameliorate diabetic alterations of peripheral nerve function through enhancing nerve blood flow (Obrosova et al., 2000). Later on, *α*1‐blocking activity was found to delay the development of STZ-induced hyperalgesia in rats (Bujalska et al., 2008 ).

Furthermore, the present study indicated noticeable histological changes of DRG neurons in diabetic rats, while carvedilol amended such pathological changes in a dose-related manner. It is worthy noted that carvedilol dose (10 mg/kg/d) resulted in less distinct pathological changes than those observed at its lower dose (1 mg/kg/d). These histopathological findings could be correlated with the biochemical assessment of NGF in DRG homogenate. The concentration of NGF in DRG homogenate in STZ-induced diabetic rats was extremely increased compared to control group. This result is in agreement with previous study showing that the level of NGF doubled in the DRG of diabetic rats (Schmidt et al., 2000). On the other hand, carvedilol had the ability–in a dose-related manner–to lessen the abnormal elevation of NGF content in DRG homogenate, compared to the diabetic group. Interestingly, carvedilol higher dose (10 mg/kg/d) was able to obliterate the increase in NGF concentration to the extent that no significant change from the control group existed.

In fact, NGF is a member of the neurotrophic family responsible for development and survivals of sensory neurons in DRG and sympathetic neurons by acting on tyrosine kinase A (TrkA) receptor (Khan and Smith, 2015). However, in addition to its trophic effect, NGF enhances the sensitization of nociceptors in case of peripheral tissue injury (Aloe et al., 2015). Several lines of evidence indicate that the NGF induces pain by upregulating the nociceptors receptors and mediators. NGF upregulates gene expression of several nociceptive receptors and mediators including sodium channels (Benn et al., 2001), calcitonin gene-related peptide (Price et al., 2005), substance P (Lindsay and Harmar, 1989; Skoff and Adler, 2006) and transient receptor potential vanilloid 1 (TRPV1) (Eskander et al., 2015). Besides, an *in-vivo* study of a skin nerve preparation showed the same sensitizing effect of NGF on nociceptor (Stucky et al., 1999). Interestingly, injection of NGF into healthy human skin can produce localized pain and hyperalgesia that develops within minutes (Petty et al., 1994), suggesting a sensitizing effect on nociceptors at the injection site. Moreover, the levels of NGF mRNA and protein are increased in several human pain disorders, especially in the context of inflammation as in diabetes (Denk et al., 2017). It is worth to mention that there are preclinical data for the efficacy of anti-NGF in rodent neuropathic pain models (Dos Reis et al., 2016). In animal studies, it has been shown that exogenous NGF induces longer lasting mechanical and thermal hyperalgesia (Lewin et al., 1994; Eskander et al., 2015). Moreover, an antihyperalgesic effect resulting from local or systemic NGF neutralization has also been widely shown. Local administration of an antiserum anti-NGF could prevent thermal and mechanical hyperalgesia-induced by an NGF injection into the rat paw (Gould et al., 2000). Interestingly, NGF was reported to induce facial heat hyperalgesia and play a role in trigeminal neuropathic pain in rats, representing a potential therapeutic target for management of orofacial condition (Dos Reis et al., 2016). Moreover, NGF or TrkA receptors blockade in rodent models of peripheral neuropathic pain resulted in significant antihyperalgesic effects (Ugolini et al., 2007; Wild et al., 2007). Likewise, experimental rats were immunized against mouse NGF, a procedure that caused an autoimmune depletion of NGF, substance P levels were reduced in the DRG, spinal cord, and skin by approximately 65% (Schwartz et al., 1982).

## Conclusion

This study provides evidence for the neuroprotective effect of carvedilol against *in vivo* STZ-induced DN, which could be explained–at least partly via its antioxidant effect and ability to decrease NGF concentration in DRG. Nonetheless, further studies are warranted to investigate the molecular mechanisms of neuroprotective effect of carvedilol on sensory neurons and intracellular signaling with respect to antioxidant and *α*-blocking activities. In addition, human clinical trials are needed to assess the efficacy of carvedilol on DN.

## Data Availability

The raw data supporting the conclusions of this article will be made available by the authors, without undue reservation.
